# Ethics of Telerehabilitation in Supportive Cancer Care Through a Responsible Innovation Lens

**DOI:** 10.2196/94505

**Published:** 2026-10-22

**Authors:** Maria C Lorenz, Matthew Sample, Alberto J Alves, Jana Müller, Karen Steindorf, Yvonne Wengström, Eva M Zopf, Anne M May, Martijn M Stuiver

**Affiliations:** 1 Centre for Ethics and Law in the Life Sciences, Leibniz University Hannover, Hannover, Lower Saxony, Germany; 2 Independent Researcher, Boston, MA, United States; 3 OncoMove, Associação de Investigação de Cuidados de Suporte em Oncologia (AICSO), Vila Nova de Gaia, Portugal; 4 Research Center in Sports Sciences, Health Sciences and Human Development, University of Maia, Maia, Portugal; 5 Exercise Oncology Research Group, Department of Medical Oncology, Heidelberg University Hospital, Medical Faculty Heidelberg, Heidelberg University, Heidelberg, Germany; 6 National Center for Tumor Diseases (NCT), NCT Heidelberg, a partnership between DKFZ and the Heidelberg University Hospital, Heidelberg, Germany; 7 Division of Physical Activity, Cancer Prevention and Survivorship, German Cancer Research Center (DKFZ) and National Center for Tumor Disease (NCT), Heidelberg, Germany; 8 Karolinska Comprehensive Cancer Center, Karolinska University Hospital, Stockholm, Sweden; 9 Department of Neurobiology, Care Sciences and Society, Karolinska Institutet, Stockholm, Sweden; 10 Mary MacKillop Institute for Health Research, Australian Catholic University, Melbourne, Victoria, Australia; 11 Cabrini Cancer Institute, Cabrini Health, Melbourne, Victoria, Australia; 12 Julius Center for Health Sciences and Primary Care, University Medical Center Utrecht, Utrecht University, Utrecht, The Netherlands; 13 Division of Psychosocial Research and Epidemiology, The Netherlands Cancer Institute, Plesmanlaan 121, Amsterdam, 1066 CX, The Netherlands, +31 20512 2480

**Keywords:** digital health, eHealth, ethics, ethical issues, telerehabilitation, remote exercise

## Abstract

In recent years, remote exercise services (“telerehabilitation”) have been increasingly explored and implemented, including in supportive cancer care, on the promise of greater convenience, access independent of geographic location, reduced infection risk, and cost savings. Ethical commentary on telehealth in general is substantial, but sustained discussion of questions specific to telerehabilitation remains comparatively sparse. As contributors to PREFERBALE-II, a European Union–funded project evaluating live-remote exercise for people recovering from cancer treatment, we sought practical guidance on how to deliver such services responsibly, and found little to work with. This prompted this viewpoint, which sets out our own position as researchers and clinicians engaged in this work. We draw on a purposive sample of professional guidance documents, which were assembled through our professional networks and a supplementary web search, and on a narrative reading of qualitative studies chosen to represent accounts of benefits as well as burdens. We read these documents through an analytical lens of responsible innovation. We make 3 arguments. First, the ethical content of current telerehabilitation guidance is largely confined to the products and processes of innovation, covering safety, privacy, consent, and compliance, while the question of purpose, that is, whether, for whom, and why remote rehabilitation should be offered at all, is left largely unasked. Anticipation appears only implicitly, and reflexivity, inclusion, and responsiveness barely at all. Responsibility is repeatedly delegated to individual professionals without commensurate structural support. Second, the situated experience of users and professionals forms part of the ethics of telerehabilitation and is not merely data to be assessed. Reported benefits and burdens depend on social context, including age, digital literacy, connectivity, caregiver availability, language, and sensory or cognitive impairment, so an equity claim made for the modality in general may not hold for the individual patient. Third, the ethics of telerehabilitation consequently has to be institutionalized across 3 overlapping domains: the interpersonal and experiential quality of remote care, the legal and institutional conditions of delivery, and the positioning of telerehabilitation within health systems. We address these arguments primarily to rehabilitation professionals and the associations and standard-setting bodies that issue guidance for them, and secondarily to digital health and ethical, legal, and social implications researchers, policymakers, and health-system decision-makers who commission or implement such services. We offer an agenda for a broader ethics conversation across professional societies, curricula, and affected publics, together with observations from a consortium put forward as hypotheses for study.

## Introduction

In recent years, there has been a surge in the exploration and implementation of live remote exercise services (telerehabilitation), including within cancer care. Before the COVID-19 pandemic and the associated demand for remote care modalities, telehealth and telerehabilitation services were already the focus of research and development efforts, from randomized controlled trials (RCTs) [1] to economic analyses [2]. In some contexts, certain prepandemic efforts were promoted not as a way to improve quality of care for patients and clients but rather as a means to increase profitability or reduce costs in health systems [3,4]. Cost is not, in itself, a suspect motive. For patients, avoiding travel, parking, and the time consumed by journeys or regular commuting can be real benefits, and for health systems, savings could potentially fund other services. However, innovation in health care raises further considerations, among them quality of care, quality of health outcomes, and equitable access, to name a few. These are ethical questions as well as practical ones, and their relevance outlasts the circumstances that made remote care urgent. Looking beyond the pandemic setting, what must we do to provide these services in a responsible and ethical way?

This challenge is multifaceted and salient for the authors of this viewpoint. We contribute to the European Union (EU)–funded PREFERABLE-II project [5], a consortium of European and Australian researchers, which aims to evaluate the value and implications of live-remote exercise (ie, a key component of telerehabilitation) for people recovering from cancer treatment. While a large-scale RCT assessing (cost-)effectiveness is at the heart of the project, we also aim to advance an informed, multisector dialogue about the ethical, legal, and social implications (ELSI) of telerehabilitation as a new technologically mediated care modality.

As a viewpoint, this paper maps and illustrates the ethical landscape of telerehabilitation without surveying it. Our focal case is the synchronous, videoconference-based form of this modality. Asynchronous apps and recorded programs appear only where the issue at stake is not specific to that format. Furthermore, AI-assisted and sensor-based rehabilitation, the wider governance of digital health, cybersecurity, and cross-border digital health regulation, beyond the professional practice questions raised here, lie outside our scope.

Previous reviews of the ethics of eHealth and telemedicine (ie, principles not specific to telerehabilitation) highlight a range of principles that need to be considered, including autonomy, trust, privacy, dignity, fairness, beneficence, nonmaleficence, justice, and, of course, the priority of health care quality over the interests of technology vendors [6-10]. Prescriptions for “ethics” of telehealth therein are often limited to preventing conflicts of interest and ensuring informed consent, leaving the rest up to the health care provider. As some have already noted, the ethics of telehealth remains underresearched empirically, especially regarding users’ perspectives [6] and overall impact on quality of care [9]. This shortfall seems even more apparent in the specific area of telerehabilitation.

The gap, in our view, is not only empirical. The most pressing questions in the ethics of telerehabilitation are not only retrospective and risk-based (eg, “Is a given service safe and compliant?”) but prospective (eg, “Should the modality be offered, for whom, and why?”). We therefore adopt responsible innovation as a lens to understand this technology-mediated modality, reframing responsibility as the prospective and collective stewardship of innovation in the present rather than the after-the-fact allocation of liability [11]. Accordingly, the responsible innovation approach would recommend a review of the full range of concerns implied by “ethics of telerehabilitation,” including not only mainstream ethical principles like informed consent but also the societal impacts of the technology-mediated approach. Following Stilgoe et al [11], we distinguish 3 levels at which ethical questions can be posed: questions about the products of innovation (eg, its risks, benefits, and their distribution), its processes (eg, how standards are drawn up and who takes part), and its purposes (eg, why a modality is pursued, in whose interest, and against which alternatives). Conventional health-professional ethics largely addresses the first 2 levels. Responsible innovation retains these but extends analysis to the often neglected level of purpose, through 4 integrated dimensions: “anticipation,” “reflexivity,” “inclusion,” and “responsiveness” [11]. This means that innovation processes need to integrate active consideration of consequences, reflect on implicit and explicit values, include participation of diverse publics and stakeholders, and then actually respond to those inputs. These requirements are motivated not only by the practical need to adapt technology to its social context but also by the demands of democratic participation in most societies. Typically, participation of publics is restricted to influencing “how” (eg, how to obtain consent, how to choose a video app) rather than “why” a technological change is being implemented [12]. These 4 dimensions structure the sections that follow.

Given this background, we aim to generate a high-level viewpoint on the current state of telerehabilitation ethics and argue 3 things: First, the ethical content encoded in current guidance on telerehabilitation remains confined to the product and process levels of responsible innovation. The question of purpose—whether, for whom, and why remote rehabilitation should be offered—is left largely unasked. Second, the lived experience of users and professionals is itself part of the ethics of telerehabilitation and is not merely data to be assessed. Listening to what users and professionals actually experience turns two of the framework’s dimensions, anticipation and inclusion, from abstract principles into something that can guide practice. Third, the ethics of telerehabilitation, therefore, have to be institutionalized across these domains: the interpersonal and experiential quality of remote care, the legal and institutional conditions of delivery, and the positioning of telerehabilitation within the health system, where the central task is to keep telerehabilitation as an additional option that broadens access, rather than letting it become a primarily cost-driven replacement for in-person care. We address these arguments primarily to rehabilitation professionals, professional associations and standard-setting bodies, and, secondarily, to digital health and ELSI researchers, policymakers, and health system decision-makers who commission or implement telerehabilitation services.

## Professional Association Guidelines

### Overview

Early in the PREFERABLE-II project, we sought to understand emerging professional norms and rules regarding the ethics of telerehabilitation, as published in professional guidelines and statements that could inform our own practice. To assemble examples, we (1) contacted the Europe Region of World Physiotherapy [13], (2) approached opinion leaders in oncology rehabilitation, identified through their standing and publication record in the field, and (3) conducted a supplementary Google search for additional publicly accessible documents. This yielded sources from Australia, Canada, Costa Rica, Latvia, and the United States. The selection is illustrative and, in keeping with the aims of a viewpoint, deliberately nonexhaustive [14]. It is meant to give an impression of how socioethical aspects are already taken into account and where there may be room for more detailed discussion, not to catalogue all available guidance. Our observations should therefore not be read as characterizing telerehabilitation guidance globally. The underlying ethical questions are likely to be shared, but the answers given to them will differ with digital, regulatory, and infrastructural conditions, and with the population served, including its demographic profile and the cancer types involved.

### Recurring Ethical Principles in Professional Guidance

The guidance we consulted ranged from formal guideline documents issued by professional associations and regulatory bodies, setting out structured principles, to more informal, often pandemic-era material such as Q&As and online brochures. We concentrate here on formal guidelines, aiming for more comparability across settings. Most of the illustrative set of formal guidelines (Multimedia Appendix 1) were issued in the first months of the pandemic, which helps to explain some of the gaps we discuss below. They vary in scope and target audience, ranging from implementation steps and technical requirements to education, clinical principles, and ethics, and from documents for practitioners to guidance on supervising physiotherapy students. Overall, we observe that a small set of ethical or socioethical principles and issues is consistently cited within the professional guidelines discussed in this viewpoint (Table 1). These include respect for autonomy and informed consent, which encompass comprehensive information including the opportunities and risks of remote services, as well as compliance with formal ethical and legal processes. Furthermore, there is some focus on data protection, privacy and confidentiality, safety, and, when relevant, local procedures, such as referral. Perhaps unsurprisingly, this list does not differ substantially from the principles that are often proposed for the ethics of health care in general.

**Table 1.** Commonly cited ethical principles in telerehabilitation guidance and example quotations.

Principle	Illustrative quotes
Compliance (legal/ethical)	*Organizations and professionals shall be aware of and comply with any applicable laws, regulations, statutes, and/or telerehabilitation-related policies and adhere to professional codes of ethics.* [15]
Autonomy and informed consent	*Organizations and/or professionals shall inform clients of their rights and responsibilities when receiving rehabilitation and habilitation services through telerehabilitation, including their right to refuse or discontinue services.* [15]
Data protection, privacy, and confidentiality	*The physiotherapist must ensure compliance with all privacy and security requirements both during tele-rehabilitation sessions and when in contact with the patient through other electronic means, such as arranging appointments via email.* [16]
Safety	*The physiotherapist must have a safety protocol in place in the event of an emergency or adverse event.* [16]
Location and referral processes	*Even under current conditions, it is preferable for physiotherapists to maintain usual referral processes. It is preferable for physiotherapists with advanced skills to support local physiotherapists, rather than be a substitute for them, unless this is mutually agreed and the impact on continuity of care for the client has been considered.* [17]

In some documents “ethics” is understood as awareness of and adherence to general ethical standards, that is, compliance with a preexisting code of ethics or conduct, either of the association itself or of an overarching institution. In Australia, for example, one guideline refers to the code of conduct of the Australian Health Practitioner Regulation Agency [17,18]. Compliance with legal processes is mentioned just as frequently. However, legal challenges are not limited to existing frameworks: emerging remote services raise novel questions, especially when offered across jurisdictions.

Respect for autonomy, beyond consent, plays an important role in practice (ie, ensuring that client participation in remote care is informed, voluntary, and empowering) and in the consulted guidelines. One Canadian guideline advises physiotherapists to “[...] augment routine informed consent processes [...],” including that clients are made aware of limitations of telerehabilitation in contrast to in-person training and consent to its technical aspects, although beyond the impracticability of hands-on examination those limitations are not specified [16]. A Latvian guideline flags data-protection requirements, recommends plain and comprehensive language, and calls for clarification of possible costs [19]. By contrast, others state that consent processes do not differ from in-person rehabilitation [20].

Importantly, the standard these documents invoke, for example, “[a] voluntary decision [...] made with knowledge and understanding of the benefits and risks involved” [18], can be difficult to meet where evidence on the risks and benefits of telerehabilitation is unavailable or not contextually suitable for a given patient, a difficulty compounded by the narrower informational basis of remote assessment, to which we return below. Reliance on for-profit telecommunications and technology suppliers poses threats to privacy, data security, and the continuity of services, as commercial parties may raise prices or reduce quality or security standards. Taking part from home further exposes intimate spaces, living situations, and household members to potentially being viewed, logged, and recorded. The guidelines often conflate data protection, privacy, and confidentiality, yet address all three, most extensively in an Australian list of privacy measures [17] and in Canadian documents on cross-provincial practice [16] and confidentiality [20].

One American document is, in this respect, the exception: it separates administrative, clinical, technical, and ethical principles and asks that organizational values and ethics be built into telerehabilitation policies and procedures instead of being left to the awareness of the individual professional [15].

Safety constitutes a minimum standard for any technological platform and a core value in health professions (“do no harm”), encompassing physical as well as psychological and emotional safety, and it features prominently in the guidelines. It covers technical aspects such as instructions for dealing with difficulties or emergencies, setting up the video call, and arranging the room safely. It includes the judgment of when not to offer telerehabilitation at all, for instance, where the client is likely to fall, has a disability the service does not accommodate, or has no caregiver available to assist. Yet, these considerations are typically only listed, leaving rehabilitation professionals to develop concrete solutions and to define what adequate remote support, training, and a mutual attitude of trust would require [17,21].

Some guidelines additionally highlight the issue of location: where the professional is, how far the service area extends, and whether referral processes remain standardized or, for services offered across a border, require permission at the client’s location [17]. Where telerehabilitation is not already regulated within a region’s health system, the ability to care for *any* client with an internet connection could circumvent existing norms of referral and service. That is a particular challenge in highly regulated professions and in transnational contexts like the EU, where an online standard of practice would have to be harmonized institutionally, culturally, and legally across jurisdictions.

### What the Guidelines Do and Do Not Ask

Reading these documents through the lens of responsible innovation, the ethical content of these guidelines is unevenly distributed and, by design, covers only part of what ethical telerehabilitation requires. Safety and data protection correspond most closely to product concerns, for example, the identification and mitigation of risks and harms, while consent, compliance, and referral processes are largely process concerns, specifying how services should be conducted and how standards apply [11]. Both are addressed, if unevenly. Purpose-related aspects, by contrast, are markedly underdeveloped, which is unsurprising: guidance of this kind is written to help practitioners deliver a service safely, not to ask whether it should be delivered at all.

Turning to the 4 introduced dimensions, anticipation appears only implicitly, in scattered cautions about when not to offer remote care. Reflexivity, the public examination of the assumptions and values built into the modality itself, is largely absent, as guidance tends to treat telerehabilitation as a settled good to be implemented safely rather than as a choice to be scrutinized. Inclusion and responsiveness are addressed least of all: the consulted documents rarely ask who should participate in deciding whether and how telerehabilitation is adopted, and they build little institutional capacity to adjust course in response to what users and participants actually experience.

Instead, responsibility for realizing ethical practice is repeatedly delegated to individual rehabilitation professionals, with subsidiarity as the implicit norm. Remote assessment, digital communication, and risk management at a distance are distinct competencies, set out in detail elsewhere [22], and the documents assume them without defining or resourcing them. Such delegation is a workable division of labor where structural support and supportive steering capacity that responsible innovation regards as essential are supplied elsewhere in the system [11]. At present, they largely are not. That inclusion and responsiveness go mainly unaddressed across the guidance landscape is, therefore, a substantive finding about telerehabilitation governance and not a shortcoming of any individual document.

None of this is peculiar to the documents we consulted. The same framing of telerehabilitation as a settled good, and the same reliance on individual practitioners to manage its tensions, are already rehearsed in professional training. In Canada, for example, rehabilitation curricula expect providers to balance advocacy for novel technologies (as a somewhat intrinsic good) with clients’ unique needs, two things that may not be straightforwardly aligned [23].

Within their remit, the guidelines usually flag a subset of important ethical issues, and across them, we noted a fundamental assumption that deserves further emphasis here: both telehealth and telerehabilitation are understood as *supporting* conventional interventions and not replacing them. Some guidelines put this more strongly, asking that telerehabilitation be equally or even more effective than the in-person approach, which does not mean or imply a replacement of in-person services [19,21]. This is not merely caution toward a new technology. Keeping both modalities available is what allows patients and professionals to choose the format that fits a given health concern, and a choice between options is a prerequisite for consent and shared decision-making to work at all.

Our approach also surfaced gaps that need more attention. Comprehensive information, in advance and during remote care, is stressed as necessary for informed consent and respect for autonomy, but rehabilitation professionals may need more concrete guidance to actually meet these expectations. What are the documented risks and benefits of remote care? And what is the role of shared decision-making in informed consent for this new remote care modality? Answering these questions is not the task of any single association, which is precisely why the more foundational questions of why and when telerehabilitation should be used need to be taken up as part of a broader dialogue.

## The Key Ingredient: Perspectives From Users and Rehabilitation Professionals

From a responsible innovation perspective, we realized that the key ingredient for developing a mature ethics of rehabilitation is deeper engagement with user and professional experience. Engaging with feedback from our clients and patients, as well as published qualitative studies, provides us with a primary resource for two of the framework’s dimensions. It supports anticipation, understood not as the prescription of outcomes but as the surfacing of the plausible positive and negative experiences a modality makes possible, and inclusion, by bringing the situated perspectives of users, carers, and professionals to bear on the prior question of whether and how remote care should be offered. Altogether, this allows for a more comprehensive ethical reflection, especially in terms of posing the foundational “why” questions in this respect.

Our engagement with the qualitative literature was narrative and illustrative in intent: studies were selected purposively by the author team to represent the range of reported experience, including accounts of benefit and accounts of burden. The studies cited therefore serve as demonstrative instances that make the ethical questions concrete.

When the technology works as intended, many users see it as a positive option and a source of added well-being. Reported motivators include the convenience of staying at home, for example, to fulfill care responsibilities or to avoid difficult commutes to the clinic, broader access to meaningful human contact that might otherwise be inaccessible, and, in the case of longer treatment, a much-needed sense of support and continuity of care. Interviewees in one study called this “closeness at a distance” [24-26].

These examples of successful use of telerehabilitation show that remote care can align with professional rehabilitation ethics, especially those related to trust, informed consent, and autonomy, but success requires preparation, as one interviewed physiotherapist makes clear:

*[T]ell patients what to expect for the virtual visit [...] a module or link on how to make the most out of the visit. Giving them steps like arriving five minutes before the visit, test the camera and audio, wear clothes for exercise, bring any equipment they have. Also, continuing to communicate the expectations of the visit and that there are going to be limitations for what we can assess virtually.* [27]

Crucially, not every context for telerehabilitation use is conducive to effective implementation, especially in the setting of supportive cancer care. Responsible innovation of telerehabilitation means addressing contextual factors that are not evenly distributed, such as clinical resources, internet availability, and software and camera technology [26,28-33]. Other significant factors include user age and digital literacy, choice of video platform, caregiver involvement, language and the need for an interpreter, institutional, and infrastructural readiness, cultural norms related to privacy, lack of touch, and interpersonal relationships.

In supportive cancer care, the distribution matters in both directions. The population is disproportionately older [34] and frequently living with comorbidity [35] or mobility limitations [36], which are precisely the conditions that make traveling to a clinic difficult, so for some patients remote delivery removes a barrier that in-person care imposes. The same population is also less likely to report high digital literacy [37,38] or a reliable connection [38], so, for others, the format substitutes one barrier for another. Sensory and cognitive impairments sharpen the point: platforms without captioning, screen-reader compatibility, or adjustable pacing exclude by design, and patients who need an interpreter face a service in which a third participant must be accommodated on a platform that is rarely built for one. Where such accommodations are unavailable, telerehabilitation does not extend access; it redistributes it, and an equity claim made for the modality in general can be false for the individual patient in front of the professional.

Improperly addressed, contextual factors become a narrative of clients’ rejection of technology [31]:

*...Because it seemed like a lot of trouble to do it online. We thought that she would not understand the program and we would not know how to do it...*

[Son, 56 years, control group]

*...We work and did not have time to do the exercises with her. Also my mother is not a person with much will and it would be very difficult to pay attention to exercises on the computer...*

[Daughter, 54 years, control group]

Qualitative work with rehabilitation experts suggests that remote delivery does not dissolve the therapeutic alliance but redistributes it: verbal guidance substitutes for manual correction, nonverbal cues are constrained by camera position and image quality, and touch, where it is needed at all, is applied by a relative under instruction [39]. Two further shifts follow from the supervised character of rehabilitation. Movement quality, gait, balance, and pain behavior are judged through observation and physical examination, and what the camera frame narrows should be stated to the client as part of consent instead of being absorbed silently into professional judgment. Remote delivery can also shift the immediate capacity to respond to a fall or another adverse event from the professional to the household, so, where fall risk is material, the presence of another person may become a precondition of the service and not an optional convenience [22,39].

Most importantly, in line with guideline recommendations, most qualitative studies report a preference for telerehabilitation as a *supplement*, not a replacement, for in-person care. Such a preference follows naturally from the fact that the *need for* and *effectiveness of* telerehabilitation are both entangled with the specific social context of its use.

Read against the 4 dimensions of responsible innovation, this material hints at aspects the guidelines leave undone. It supplies raw material for anticipation, since it surfaces the plausible harms and benefits a modality makes possible before they are formalized as risks. It is a route to inclusion, since it is where users, carers, and frontline professionals speak. Additionally, it makes responsiveness testable, since a service that never revises its design in the light of such accounts is not responsive in any meaningful sense. What this material cannot supply on its own is reflexivity, which requires the professions themselves to examine the assumptions built into remote delivery.

## Observations From Our Own Practice

This section takes up that task in part for our consortium. Designing the PREFERABLE-II trial required us to decide how remote exercise would be delivered, and those decisions raised ethical questions we had not completely anticipated. We report what our discussions surfaced as situated experience of the kind this viewpoint treats as ethically informative, and we offer them as hypotheses for study rather than findings about frequency.

One of the issues discussed was the fostering of healthy group dynamics. Group-based telerehabilitation offers an opportunity to learn from peers and experience connectedness. However, the telerehabilitation setting prohibits “physical” peer-interaction, such as collaborative exercises. Also, it might make it more difficult to pick up the subtle forms of communication required for effective peer-to-peer and patient-rehabilitation professional interactions. Members of our consortium have also suggested the need for clear guidance on liability for accidents, especially when seeing clients outside their normal in-person jurisdiction, as well as the question of whether consent needs to be obtained from clients’ household members, who may not want to be recorded. Fairness, too, has become a logistical question, as we plan how to provide space, internet bandwidth, and equipment to participants who may lack those resources [22], while also considering whether functional limitations or safety concerns make live, remote exercise unsuitable.

Some concerns appear less pressing in practice, at least in some of the work of PREFERABLE-II partners. Initial unease among trainers about seeing clients’ homes often shifted into rapport, as it somehow creates a sense of closeness for them, even though they know the patients only online. Postpandemic familiarity with online interaction may also have eased adoption for potential users. Furthermore, some of our colleagues report that the absence of touch has been less of an obstacle than expected. In a trainer survey, most exercise components were rated as equally or more-effective when delivered live-remotely, though 52% and 71% judged resistance and agility training less effective [40]. Some suggest the format may support performance because trainers demonstrate the exercises more often and the camera naturally provides direct feedback.

## Toward Ethics of Telerehabilitation: 3 Priority Domains

Telerehabilitation has not yet generated a dedicated ethics literature on area-specific best practices. To our knowledge, there is no scholarly or professional consensus on what “ethical” or “responsible” telerehabilitation requires in concrete terms. Within the PREFERABLE-II project, we expect to develop our own methods to address the positive and negative potentials of remote care, as well as to answer socioethical questions. However, we believe these dimensions of practice also deserve broader consideration in rehabilitation research streams and in the development of more concrete evidence-based professional guidelines globally.

From our perspective, priorities span 3 overlapping domains, each with its own addressee. These are the sites at which the question of purpose—whether, for whom, and why remote rehabilitation should be offered—is actually settled (Figure 1).

**Figure 1.**
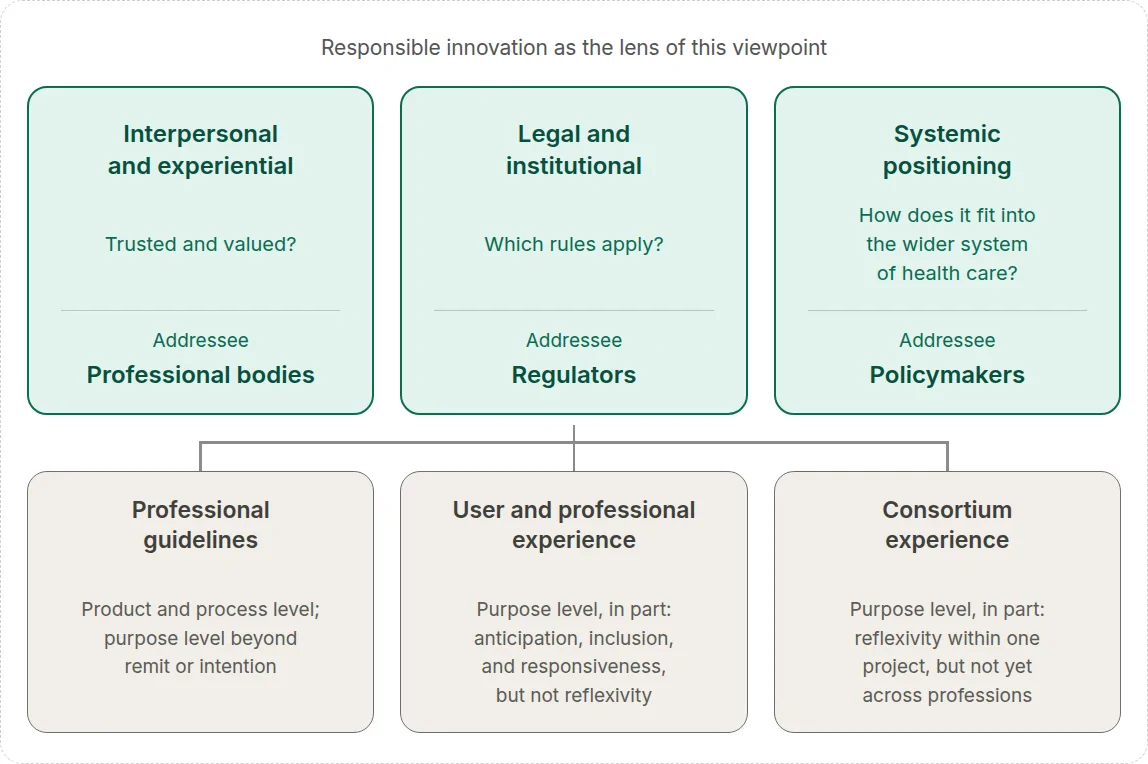
Suggestions for discussing the ethics of telerehabilitation [11].

First and most obvious are the interpersonal and experiential dimensions of practice, for example, whether clients and professionals trust the modality, whether care goals are met, and whether professionals feel capable and well-equipped. Established principles such as safety and consent apply here as they do in person, but they are harder to satisfy at a distance, and the immediate response to an adverse event lies in specific protocols and potentially with the household. This is why the question of whether remote delivery suits a given patient is a judgment about an individual person and not about a service line. It weighs accessibility, digital skills, the risks of supervised exertion at a distance, and the patient’s own preference once the limits of the format have been explained. That means it belongs to the patient and the professional together and not to the organization commissioning the modality. Professional societies and rehabilitation curricula would correspondingly need to treat remote assessment, verbal guidance, and risk triage at a distance as examinable competencies and not as improvisations.

Second, and less obvious, are the legal and institutional challenges: does the service comply with relevant institutional norms and (inter-)national regulations? Who will provide training and regulatory support in fully adapting each instance of telerehabilitation to its context? Because of the technologically mediated interaction, shifting laws concerning data handling and digital services are likely to be invoked, and cross-border services become possible, although legally questionable. Associations and regulators would need to state what applies across jurisdictions, what liability attaches to an adverse event in a patient’s home, and whether the consent of household members is required.

Third, there is the systemic domain spanning questions of innovation within the health system in general. Was the approach developed with social context in mind, reflexively, and with opportunities for user input? How does it fit into existing health care offerings, and with what alternatives? Perhaps the greatest potential challenge at this societal level is preventing the premature replacement of in-person care and leaning more toward the availability of hybrid approaches, fully respecting the individual patient’s needs. Because telerehabilitation is defended here as one further option on the menu and not as a substitute, exclusion from it is not exclusion from care and no patient should be required to use it. That defense holds only for as long as the in-person route remains open: where it is withdrawn, those who cannot use the remote format lose access altogether. Another practical notion of this domain covers the reimbursement aspect. Where remote sessions are unbillable or reimbursed below in-person rates, provision depends on the goodwill of individual practices and on project funding of the kind that supports our own work. Where they are reimbursed more favorably the incentive may run toward substitution irrespective of what a given patient needs. The ethical question is therefore not whether telerehabilitation saves money but who books the savings and who carries the losses. Policymakers and regulators would accordingly need to decide the substitution question explicitly when setting reimbursement rules, so that a modality reported as a preferred supplement is not adopted as a replacement by budgetary default. A detailed health economic analysis within the PREFERABLE-II trial will show whether savings arise and where.

Looking ahead and beyond research, the ethics of telerehabilitation should be seen as a multisector conversation. Inclusive dialogue and iterative policymaking should align innovation with public needs. On that note, this viewpoint itself falls somewhat short of the inclusion it asks for. It was developed by researchers and clinicians without the direct involvement of patients or carers, whose perspectives would enrich, and, in places, might revise, the priorities we identify. Although there is no single organization or discipline that will be wholly responsible for realizing ethical telerehabilitation, the mark of a robust ethics discourse is the ability to pragmatically bring the full range of concerns to the awareness of multiple stakeholders. When read across the 3 domains, the 4 dimensions of purpose stand or fall together. Anticipation and inclusion without responsiveness leave what users report unanswered, and none of the 3 counts for much without the reflexivity that would have the professions examine the assumptions built into remote delivery in the first place.

To this end, the ethics of telerehabilitation should start with tailored research into how to bridge the gap between rehabilitation guidance and the specific problems of remote care, and work toward the institutionalization of ethics conversations across professional societies, rehabilitation curricula, interdisciplinary conferences, and affected publics. While our viewpoint is still preliminary in scope, we hope it may inspire future work in these directions.

## Supplementary material

10.2196/94505 Multimedia Appendix 1 Overview: illustrative set of professional guideline documents.

## Abbreviations

ELSI: ethical, legal, and social implications
EU: European Union
RCT: randomized controlled trial
