# Supplementary material for: Ethics of Telerehabilitation in Supportive Cancer Care Through a Responsible Innovation Lens
Source: J Med Internet Res. 2026 Oct 22;28:e94505. doi: 10.2196/94505 (PMC13690332; doi:10.2196/94505)
Supplement: Supplementary file 1 [file jmir-v28-e94505-s001.docx]

**Table S1 – Overview: Illustrative Set of Professional Guideline Documents^[1]^**

|  | **Professional Association** | **Country** | **Title** | **Publishing Date** | **Intended Audience** | **Scope** | **Covered Ethical Domains** |
| --- | --- | --- | --- | --- | --- | --- | --- |
| **1** | American Telemedicine Association | United States | “American Telemedicine Association’s Principles for Delivering Telerehabilitation  Services”[1] | November 2017 | Practitioners,  Rehabilitation Professionals,  Professional Associations,  other Organizations | 7 pages  Content:  “The content in this document addresses general administrative, clinical, technical and ethical principles and not specific practice guidelines for implementing telerehabilitation services.” | - Adherence with regulations, telerehabilitation-related policies, and codes of ethics - Information and Consent - Recommendation for having formal processes in place for resolving ethical issues - The rest of the guidelines touches on ethical aspects, too, but the above-mentioned are part of the separate “ethics” section. |
| **2** | Australian Physiotherapy Association | Australia | “Telehealth Guidelines.  Response to COVID-19”[2] | March 2020 | Profession of Physiotherapists and Practitioners | 33 pages  Content:  “The purpose of these guidelines is to give physiotherapists providing physiotherapy via real-time (synchronous) video consultation with a framework that will help ensure the safety and  quality of their practice within the context of an emergency response to COVID-19.” | - Awareness of ethical standards - Privacy - Referral processes - Safety aspects (not in the chapter on ethical aspects itself) |
| **3** | Canadian Alliance of Physiotherapy Regulators | Canada | “Tele-rehabilitation in Physiotherapy. Guidelines for Physiotherapists”[3] | 2017 | Physiotherapists | 3 pages  Content:  “Ten provincial physiotherapy regulators have entered into a Memorandum of Understanding for Cross-Border Physiotherapy. The purpose of this agreement is to enhance access to care for patients, in the best interests of patients.” | - Location and Referral Processes - Safety - Consent - Adherence with Standards of Practice and Codes of Ethics - Privacy |
| **4** | Colegio de Terapeutas de Costa Rica (CTCR)^[2]^ | Costa Rica | “Lineamientos Generales  Para El Ejercicio De La  Telesalud”[4] | April 2020 | Therapists registered with CTCR | 6 pages  Content: General guidelines for the practice of telehealth, including rehabilitation. It encompasses: definition and scope, professional responsibilities, and minimum components of service delivery. | No specific chapter on ethical aspects.   - Telehealth as Complement, not Replacement - Informed Consent - Confidentiality & Data Protection - Safety - Fair Commercial Conduct |
| **5** | Latvijas Fizioterapeitu Asociacija^[3]^ | Latvia | “Attālināta fizioterapijas pakalpojuma  sniegšanas kvalitātes kritēriji un  rekomendācijas ieviešanai praksē”[5] | April 2020 | Physiotherapists,  Physiotherapy Practices | 9 pages  Content:  Quality criteria and practical recommendations for remote physiotherapy: scope of service, informed consent and GDPR compliance, documentation, ethics and limits of professional competence, technical and training prerequisites. | - Information & Consent - Compliance with Ethical Standards/Guidelines - Safety (not in the chapter on ethical aspects itself) - Data Protection (not in the chapter on ethical aspects itself) |
| **6** | National Association for Clinical Education in Physiotherapy | Canada | “Guidelines for Supervising Physiotherapy Students Engaged in  Tele-Rehabilitation as part of Clinical Education”[6] | June 2020 | Clinical Instructors, Physiotherapy Students | 13 pages  Content: “These guidelines aim to highlight the […] [differences between in-person and tele rehabilitation physiotherapy] and intend to  provide the Clinical Instructor (CI) and student with guidance for clinical education experiences that  involve patient care using TR.” | - Consent - Confidentiality - Safety |

References

1. Richmond T, Peterson C, Cason J, et al. American Telemedicine Association’s Principles for Delivering Telerehabilitation Services. International Journal of Telerehabilitation. 2017; 9(2): 63-68. doi: <https://doi.org/10.5195/ijt.2017.6232>

2. Australian Physiotherapy Association. Telehealth Guidelines Response to COVID-19. 2020. <https://australian.physio/sites/default/files/APATelehealthGuidelinesCOVID190420FA.pdf> (accessed: 03 September 2025)

3. Canadian Alliance of Physiotherapy Regulators. Tele-rehabilitation in Physiotherapy Guidelines for Physiotherapists. 2017. <https://alliancept.org/wp-content/uploads/2016/06/Tele-rehabilitation-Guidelines-for-Physiotherapists-Public.pdf> (accessed: 03 September 2025)

4. Colegio de Terapeutas Costa Rica. Lineamientos Generales Para El Ejercicio de la Telesalud. 2020. <https://colegiodeterapeutas.cr/wp-content/uploads/2021/07/lineamientos-telesalud-1.pdf> (accessed: 03 September 2025)

5. Latvijas Fizioterapeitu Asociacija. Attālināta Fizioterapijas Pakalpojuma Sniegšanas Kvalitātes Kritēriji Un Rekomendācijas Ieviešanai Praksē. 2020. <http://www.fizioterapeitiem.lv/attachments/article/1418/Attālināta%20FT%20pak%20sniegšanas%20kvalitates%20kriteriji%20un%20rekom%20ieviesanai%20prakse%202020%201%20aprilis.pdf> (accessed: 03 September 2025)

6. National Association for Clinical Education in Physiotherapy. Guidelines for Supervising Physiotherapy Students Engaged in Tele-Rehabilitation as part of Clinical Education. 2020. <https://med-fom-clone-pt.sites.olt.ubc.ca/files/2020/08/Guidelines-for-Telerehabilitation-in-Clinical-Education-2020.pdf> (accessed: 03 September 2025)

1. This table presents an illustrative, non-exhaustive selection of guidance documents identified through a convenience search, as explained in the main text of the paper. No systematic search was conducted, and no predefined search strategy, database selection, search terms, or eligibility criteria were applied. The table is intended to illustrate the range of existing guidance and the aspects it does and does not address. It does not support claims about the prevalence, completeness, or representativeness of the guidance landscape.
2. The original document is available in Spanish only. The information and summaries given here are based on a DeepL translation.
3. The original document is available in Latvian only. The information and summaries given here are based on a DeepL translation.
